# Associations of Objectively Measured Physical Activity and Sedentary Time with the Risk of Stroke, Myocardial Infarction or All-Cause Mortality in 70-Year-Old Men and Women: A Prospective Cohort Study

**DOI:** 10.1007/s40279-020-01356-y

**Published:** 2020-10-15

**Authors:** Marcel Ballin, Peter Nordström, Johan Niklasson, Anna Nordström

**Affiliations:** 1 Department of Community Medicine and Rehabilitation, Unit of Geriatric Medicine, Umeå University, 901 87 Umeå, Sweden; 2 Department of Public Health and Clinical Medicine, Section of Sustainable Health, Umeå University, Umeå, Sweden; 3 School of Sport Sciences, UiT The Arctic University of Norway, Tromsø, Norway

## Abstract

**Objective:**

To study the associations of objectively measured physical activity (PA) and sedentary time (ST) with the combined outcome of incident stroke, myocardial infarction (MI) or all-cause mortality in older adults.

**Methods:**

*N* = 3343 men and women aged 70 who participated in a health survey between 2012 and 2017 were included. Actigraph GT3X+ accelerometers were used to measure light-intensity PA (LPA), moderate-intensity PA (MPA) and ST for 1 week. Incident cases of cardiovascular disease (CVD) in terms of stroke or MI, and all-cause mortality were identified using national registers. Hazard ratios (HR) and 95% confidence intervals (CI) were estimated using multivariable-adjusted Cox regressions.

**Results:**

During a mean follow-up of 2.7 years (0.1–5.6), there were 124 events of CVD or all-cause mortality. After adjusting for potential confounders and mediators, every 30-min/day increment in LPA was associated with 11% lower risk of CVD or all-cause mortality (HR 0.89, 95% CI 0.82–0.97), and every 30-min/day increment in MPA was associated with 36% lower risk (HR 0.64, 95% CI 0.48–0.84). Every 1-h/day increment in ST increased the risk of the outcomes by 33% (HR 1.33, 95% CI 1.14–1.56), although there was no significant association among participants who performed ≥ 30 min/day MPA (HR 1.11, 95% CI 0.82–1.50, *P* = 0.034 for interaction). None of the associations were modified by sex (*P* > 0.4 for all).

**Conclusion:**

Objectively measured LPA and MPA are each associated with lower risk of stroke, MI or all-cause mortality in 70-year-old individuals, while ST is associated with increased risk. The greatest risk reduction is observed for MPA, which also appears to attenuate some of the increased risks associated with ST.

## Key Points

**Table:** 

In this study of more than 3300 70-year-old men and women, we found that both light- and moderate-intensity physical activity were each linked to lower risk of stroke, myocardial infarction or all-cause mortality. Moderate-intensity physical activity entailed the greatest relative risk reduction, around threefold that of light-intensity physical activity.
Sedentary behavior was linked to an increased risk of stroke, myocardial infarction or all-cause mortality. However, we also found that some of the increased risks pertaining to sedentary behavior may potentially be attenuated by performing moderate-intensity physical activity.
The findings from the present study may support the development and implementation of environmental actions and interventions aiming to reduce the risk of cardiovascular disease and premature death in older adults. Essentially, the main finding could be translated into one simple take-home message that is easily communicated to the public at large: “Regardless of intensity, the more you move and the less you sit, the better”.

## Introduction

Cardiovascular disease (CVD) is strongly related to ageing [1, 2], and of all CVDs, stroke and myocardial infarction (MI) cause the majority of deaths [3]. Recently, these were also shown to increase in conjunction with the rapid population ageing [4]. However, the World Health Organization states that at least 80% of all stroke and MI may be prevented through lifestyle changes [5]. Therefore, identifying key behavioral factors that could substantially reduce the risk of CVDs including stroke and MI in older people specifically is pivotal to effectively manage the rapid growth of the older population [4, 6], and would be of interest to healthcare providers, policymakers and stakeholders considering that CVD impose a significant economic burden for the society [7, 8].

An abundant and convincing body of evidence points to the vast importance of adopting an active lifestyle to attain health benefits all the way from a cellular to a disease-specific level [9, 10]. Specifically, the positive effects of physical activity (PA) and hazards of sedentary behavior in relation to the risk of incident CVD and mortality have been demonstrated by numerous studies during the recent years [11–15]. However, until more recently, most of the research was conducted using self-reported methods for assessing PA [16], which are more inaccurate compared to objective methods due to their susceptibility to recall- and social desirability biases [17, 18]. In addition, they have limited ability to capture light-intensity PA (LPA) and sedentary time (ST) [19, 20], which is a particularly dilemma when studying older people given that most of the PA that they perform is LPA, and they spend at least 60–75% of their awake time in a sedentary state [21, 22].

Consequently, there has been a request for more studies investigating the association of objectively measured PA of different intensities and sedentary behavior with clinical health outcomes [16]. In particular, the association of LPA and ST with CVD and mortality in older people has gained increased attention; however, the findings are inconsistent [23–26]. This type of research has high clinical relevance given that there may be a misconception of the volume and intensity of PA that is necessary to reduce the risk of CVD which acts as a barrier towards PA [9, 10]. In the wake of the above, and the low adherence to the current PA recommendations [2], evidence for benefits pertaining to LPA may have a fundamental impact on PA promotion and would facilitate for older people to adopt and adhere to an active lifestyle. To this end, we investigated the associations of objectively measured PA of different intensities and ST, with the prospective risk of stroke, MI or all-cause mortality in a large cohort of 70-year-old men and women.

## Methods

### Study Design and Population

This was a prospective cohort study based on the ongoing population-based primary prevention study Healthy Ageing Initiative (HAI) conducted in Umeå, a municipality in northern Sweden with 128,901 inhabitants in 2019. HAI aims to identify traditional and novel risk factors for CVD, falls, and fractures among all 70-year-olds in Umeå. The participants partake in an extensive health examination including objective measurements of PA, body composition, and cardiometabolic risk markers among others. The participants are instructed to be in a fasting state for at least 4 h when arriving to the clinic. There, the examinations are performed by one of five research nurses supported by two chief physicians (AN and PN). To be included, one must be a citizen of Umeå municipality and at the age of 70. Recruitment is performed using population registers, and there are no exclusion criteria. Around 70% of all eligible individuals choose to participate in the HAI-study [27]. In the present study, all HAI-participants from May 2012–October 2017 with available measures of objective PA were eligible for inclusion. The HAI-study and the present study were both approved by the Regional Ethical Review Board in Umeå, Sweden (no. 07-031M with extensions).

### Assessment of Physical Activity and Sedentary Time

PA and ST were measured during 1 week of registration using Actigraph GT3X+ accelerometers (Actigraph, Pensacola, FL, USA). The accelerometer was attached to a hip-worn belt and placed at the nondominant hip of the participants. The instructions were to wear the accelerometer for 7 days at all times except for when sleeping, showering or bathing, and to be normally active during these days. The raw accelerometer data were collected at 30 Hz frequency and filtered using the standard Actigraph filter to eliminate non-human accelerations. Using Actilife software 6.11.3, the raw data were transformed into counts of movement in 60 s epoch lengths. Next, wear time validation was applied, where participants were required to have accumulated ≥ 10 h wear time/day for ≥ 4 days to be included in the analysis. Non-wear time was defined as at least 60 consecutive minutes of zero counts, with an allowance of a maximum of 2 min of counts between 0–100. Cut-points used for classification of PA intensities was based on the work by Freedson [28], classifying ST, LPA, moderate-intensity PA (MPA) and vigorous-intensity PA (VPA) as follows: ST < 100 CPM; LPA (100–1951 CPM); MPA (1952–5724 CPM); VPA (≥ 5725 CPM). Moderate-to-vigorous PA (MVPA) was calculated by summing MPA and VPA. Because not all participants wore the accelerometer for the full week of registration, adherence to the PA recommendations [16] was calculated based on daily averages, where participants with at least 30 min/day of MVPA were considered to meet the PA recommendations.

### Assessment of Other Variables and Covariates

Baseline data and selected covariates were collected in the HAI-study and through national registers. Specifically, height and weight were measured to calculate the body mass index (BMI, kg/m^2^). Blood pressure was measured using a digital automatic blood pressure device Omron M6 Comfort HEM-7221-E (Omron Healthcare, Kyoto, Japan) after a 15-min rest. Fasting blood glucose was measured using the HemoCue 201 RT system (Radiometer Medical ApS, Denmark), while blood lipids were analyzed at the accredited laboratory at the department of clinical chemistry, Umeå University hospital. Whole-body scans using a Lunar iDXA device with the CoreScan application (GE Healthcare Lunar, Madison, WI, USA) were performed to quantify visceral adipose tissue. Socioeconomic data (education, marital status, income) were collected from the registers of Statistics Sweden. Information on medications was collected from the Prescribed Drug Register, which covers all medications dispensed at pharmacies in Sweden since July 2005. Data on previously diagnosed medical conditions were collected from the National Patient Register (NPR), superintended by the Swedish National Board of Health and Welfare. The NPR covers all inpatient care in Sweden since 1987 and all secondary outpatient care since 2001.

### Ascertainment of CVD and All-Cause Mortality

The main outcome was the composite endpoint of CVD (stroke or MI) or all-cause mortality until 31 December 2017. Secondarily, CVD and all-cause mortality were analyzed separately. Incident cases of stroke and MI were collected from the NPR and using the International Classification of Diseases, 10th ed diagnostic codes I61–I64 and I21. In general, the positive predictive value (PPV) for diagnoses in the NPR is between 85 and 95%, where the PPV for diagnosis of stroke is between 69 and 99% and for MI, the PPV is 86–100% [29–31]. Data on all-cause mortality were collected using the Swedish Cause of Death Register which is complete since 1961 [32].

### Data Linkage

All HAI-data and registry data were linked together using the personal identity number that is unique and issued to all residents of Sweden. The data files were retrieved from the National Board of Health and Welfare on December 21, 2018.

### Statistical Analysis

To examine the associations between the exposures and the outcomes, Cox proportional hazard regression models were performed. The proportional hazards assumption was assessed using covariate-by-time interaction terms, and the assumption was not violated. Hazard ratios (HR) and 95% confidence intervals (CI) were estimated for 30-min/day increments in LPA, MPA and MVPA, and for 1-h/day increments in ST. Follow-up time was calculated as the number of days from participation in HAI until the first incident case of either stroke, or MI, or death, whichever came first. Following the first event, participants were censored. If no event occurred, follow-up time ended on 31 December 2017. Linearity was tested by adding squared terms for each exposure to the models. Adjustment for potential confounders and mediators was performed in multiple steps. Model 1 was adjusted for sex and accelerometer wear time. Model 2 was additionally adjusted for smoking (yes/no), marital status (married/never married/widowed/divorced), level of education (primary/secondary/post-secondary), and disposable income. In model 3, CVD-history (MI/stroke/angina pectoris) and medication (anti-hypertensives/anti-coagulants/statins) was added. In the 4th and finally adjusted model, systolic blood pressure, visceral adipose tissue, fasting blood glucose, and low-density lipoprotein cholesterol was added.

Next, using Cox regression models, the associations were investigated in subgroups according to significant risk factors for the composite endpoint of CVD or mortality identified in the main analyses. In addition, the associations of LPA and MPA with the composite endpoint were also investigated in subgroups of participants with ST < 8.8 h/day and ≥ 8.8 h/day (median split), while the association of ST with the main outcome was investigated in subgroups based on amount of MPA (0–15 min/day, 16–29 min/day and ≥ 30 min/day). Interactions were tested by adding product terms for each covariate and exposure and placing them into the Cox model. Each subgroup analysis was adjusted for sex, accelerometer wear time and other significant risk factors.

Finally, in an attempt to decrease the risk of reverse-causality bias, a sensitivity analysis was conducted. For each exposure, a Cox regression model was performed where all participants with a history of CVD were excluded, as were all participants with a follow-up time of 6 months or less. These models were adjusted according to model 4 in the main analysis, described two paragraphs above. SPSS version 25.0 (IBM, Corp. Armonk, NY) was used to perform all analyses with the significance level set at *P* < 0.05.

## Results

### Participant Characteristics

From 3618 consecutive individuals that participated in HAI from May 2012 to October 2017, 275 (7.6%) had insufficient accelerometer wear time and were excluded from the analysis. Thus, in total 3343 participants (51% female) with a mean age of 70.5 years were included in this study. During 1 week of registration, 1604 participants (48.0%) adhered to the PA recommendations of at least 30 min/day of MVPA. Mean daily wear time was 13.8 h and mean number of wear days was 6.6 days. Common diagnoses were depression (20.1%) and cancer (18.7%), while 3% and 4% had previously suffered a stroke and MI, respectively (Table 1).

**Table 1:** Baseline characteristics of the study cohort comprising 3343 men and women aged 70 who participated in the Healthy Ageing Initiative study during May 2012–October 2017

Age, years	70.5 ± 0.1
Female sex, *n* (%)	1693 (50.6)
Currently smoking, *n* (%)	199 (6.0)
BMI, kg/m^2^	26.4 ± 4.2
Visceral adipose tissue, g	1495 ± 972
Systolic blood pressure, mmHg	139 ± 17
Diastolic blood pressure, mmHg	81 ± 9
Fasting blood glucose, mmol/l	5.7 ± 1.2
Low-density lipoprotein cholesterol, mmol/	3.3 ± 1.1
High-density lipoprotein cholesterol, mmol/l	1.6 ± 0.5
Total cholesterol, mmol/l	5.5 ± 1.2
Triglycerides, mmol/l	1.3 ± 0.7
Accelerometer measurements	
ST, h/day	8.9 ± 1.4
LPA, min/day	263.6 ± 71.6
MPA, min/day	32.5 ± 24.9
VPA, min/day	0.9 ± 4.1
MVPA, min/day	33.4 ± 25.7
Adherence to PA recommendations, *n* (%)^a^	1604 (48.0)
Wear time, h/day	13.8 ± 1.34
Wear days, *n*	6.6 ± 1.0
Disposable income at age 60, 1000 Swedish kronor	245 ± 175
Education,^b^ *n* (%)	
Primary	578 (17.3)
Secondary	1356 (40.6)
Post-secondary	1445 (42.1)
Missing data, *n*	4 (0.1)
Marital status,^b^ *n* (%)	
Married	2219 (66.4)
Never married	287 (8.6)
Widowed	260 (7.8)
Divorced	577 (17.3)
Missing data, *n*	0
Diagnoses and medications,^c^ *n* (%)	
Stroke	112 (3.4)
Myocardial infarction	143 (4.3)
Angina pectoris	252 (7.5)
Diabetes	288 (8.6)
Fracture	519 (15.5)
Depression	672 (20.1)
Cancer	624 (18.7)
Anti-hypertensives	1912 (57.2)
Statins	1410 (42.2)
Anti-coagulants	1276 (38.2)

*BMI* body mass index, *LPA* light-intensity physical activity, *MPA* moderate-intensity physical activity, *MVPA* moderate-to-vigorous physical activity, *ST* sedentary time, *VPA* vigorous-intensity physical activity

^a^Calculated based on daily averages. Participants with at least 30 min/day of MVPA were considered to meet the recommendations

^b^Education and marital status recorded in the calendar year before the baseline date

^c^Prescriptions filled since July 2005

Data are presented and means and standard deviations unless stated otherwise

### Physical Activity and the Risk of CVD or Mortality

During a mean follow-up time of 2.7 years (0.1–5.6), there were 124 events of the composite endpoint CVD or mortality (stroke, 39; MI, 35; death, 50) with an incidence rate (IR) of 14.2 per 1000-person-years (PY). Addition of the squared term for each exposure did not indicate that the associations were nonlinear (*P* for nonlinearity > 0.50 for all). After adjusting for sex and accelerometer wear time, every 30-min/day increment in LPA was associated with 13% lower risk of CVD or mortality (HR 0.87, 95% CI 0.80–0.95), and 11% lower risk in the finally adjusted model (HR 0.89, 95% CI 0.82–0.97, Table 2). Furthermore, every 30-min/day increment in MPA was associated with 44% lower risk of CVD or mortality after adjusting for sex and wear time (HR 0.56, 95% CI 0.43–0.74), which was reduced to 36% in the finally-adjusted model (HR 0.64, 95% CI 0.49–0.83, Table 2). Other significant risk factors for the composite endpoint of CVD or mortality included sex, fasting blood glucose, annual disposable income, smoking, anti-coagulant medication and statins (*P* < 0.05 for all). Based on the 95% CIs, the association between MPA and the composite endpoint was significantly stronger than the association between LPA and the composite endpoint in at least model 1 and model 2 (Table 2). The associations for the exposure MVPA were similar to those for MPA (Table 2).

**Table 2:** Associations of physical activity and sedentary time with incident stroke, myocardial infarction and all-cause mortality in 3343 men and women aged 70 who participated in the Healthy Ageing Initiative study during May 2012–October 2017

Stroke, MI or all-cause mortality	No. of participants (no. of events)^a^	Per 30-min increment of LPA/day	Per 30-min increment of MPA/day	Per 30-min increment of MVPA/day	Per 1-h increment of ST/day
		HR (95% CI)	HR (95% CI)	HR (95% CI)	HR (95% CI)
Model 1	3343 (124)	0.87 (0.80–0.95)	0.56 (0.43–0.73)	0.56 (0.43–0.74)	1.43 (1.22–1.68)
Model 2	3334 (124)	0.88 (0.81–0.96)	0.62 (0.47–0.80)	0.61 (0.47–0.80)	1.38 (1.18–1.61)
Model 3	3334 (124)	0.89 (0.82–0.96)	0.65 (0.50–0.85)	0.65 (0.50–0.84)	1.34 (1.15–1.56)
Model 4	3280 (121)	0.89 (0.82–0.97)	0.64 (0.48–0.84)	0.64 (0.49–0.83)	1.33 (1.14–1.56)
Stroke or MI					
Model 1	3343 (74)	0.88 (0.78–0.98)	0.50 (0.35–0.73)	0.51 (0.35–0.73)	1.45 (1.18–1.78)
Model 2	3333 (74)	0.88 (0.78–0.98)	0.55 (0.38–0.79)	0.55 (0.38–0.79)	1.41 (1.15–1.73)
Model 3	3333 (74)	0.89 (0.79–0.99)	0.59 (0.41–0.84)	0.58 (0.41–0.83)	1.37 (1.12–1.68)
Model 4	3279 (71)	0.88 (0.78–0.99)	0.59 (0.40–0.87)	0.59 (0.41–0.86)	1.38 (1.12–1.72)
All-cause mortality					
Model 1	3343 (57)	0.83 (0.74–0.94)	0.57 (0.39–0.85)	0.57 (0.39–0.84)	1.50 (1.21–1.86)
Model 2	3333 (57)	0.85 (0.75–0.95)	0.62 (0.42–0.92)	0.62 (0.43–0.91)	1.43 (1.16–1.76)
Model 3	3333 (57)	0.85 (0.76–0.95)	0.68 (0.47–0.97)	0.68 (0.47–0.96)	1.39 (1.13–1.70)
Model 4	3280 (56)	0.87 (0.78–0.97)	0.61 (0.41–0.89)	0.60 (0.42–0.98)	1.36 (1.11–1.65)

Model 1: adjusted for sex and accelerometer wear time

Model 2: adjusted for sex, accelerometer wear time, smoking status, marital status, level of education, disposable income

Model 3: adjusted for sex, accelerometer wear time, smoking status, marital status, level of education, disposable income, MI, stroke, angina pectoris, anti-hypertensives, anti-coagulants, statins

Model 4: adjusted for sex, accelerometer wear time, smoking status, marital status, level of education, disposable income, MI, stroke, angina pectoris, anti-hypertensives, anti-coagulants, statins, systolic blood pressure, visceral adipose tissue, fasting blood glucose, low-density lipoprotein cholesterol

*CI* confidence interval, *HR* hazard ratio, *LPA* light-intensity physical activity, *MI* myocardial infarction, *MPA* moderate-intensity physical activity, *MVPA* moderate-to-vigorous physical activity, *ST* sedentary time

^a^The small difference in the number of participants and events from model 1 through model 4 are caused by missing data for one or several of the covariates added in these models

**Fig. 1:**
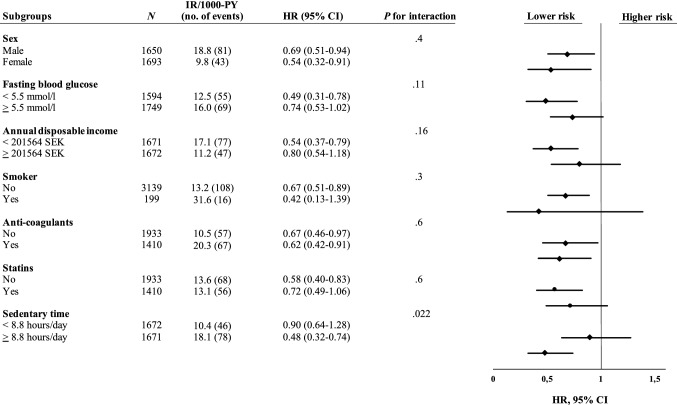
Subgroup- and interaction analyses for the association between every 30-min/day increment in MPA and the composite endpoint of cardiovascular disease (stroke or myocardial infarction) or all-cause mortality. All HRs, CIs, and *P* values were derived from Cox regression models adjusted for sex and accelerometer wear time, as well as mutual adjustment for the other significant risk factors. *CI* confidence interval, *HR* hazard ratio, *IR* incidence rate, *MPA* moderate-intensity physical activity, *PY* person years

**Fig. 2:**
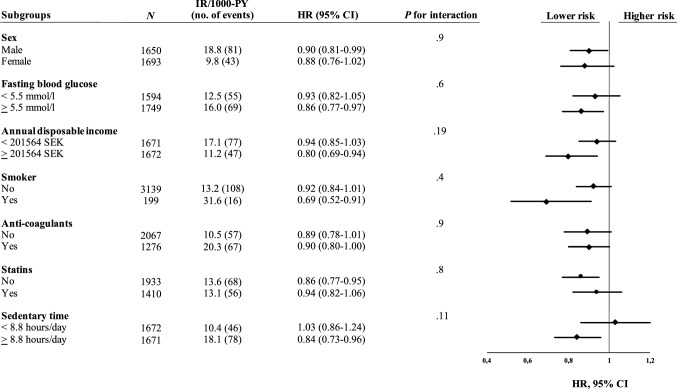
Subgroup- and interaction analyses for the association between every 30-min/day increment in LPA and the composite endpoint of cardiovascular disease (stroke or myocardial infarction) or all-cause mortality. All HRs, CIs, and *P* values were derived from Cox regression models adjusted for sex and accelerometer wear time, as well as mutual adjustment for the other significant risk factors. *CI* confidence interval, *HR* hazard ratio, *IR* incidence rate, *LPA* light-intensity physical activity, *PY* person years

Figures 1 and 2 present the results of the subgroup- and interaction analyses performed for LPA and MPA. Every 30-min/day increment in MPA was more strongly associated with lower risk of CVD or mortality in participants with above median ST/day (HR 0.48, 95% CI 0.32–0.74) compared to participants below median ST/day, where no significant risk reduction was observed (HR 0.90, 95% CI 0.64–1.28) (*P* = 0.022 for interaction, Fig. 1). A similar pattern was observed for every 30-min/day increment in LPA (HR 0.84, 95% CI 0.73–0.96 vs HR 1.03, 95% CI 0.86–1.24), although the interaction was not statistically significant (*P* = 0.11).

### Sedentary Time and the Risk of CVD or Mortality

**Fig. 3:**
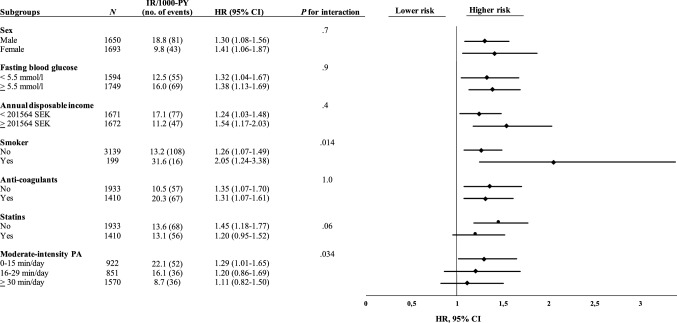
Subgroup- and interaction analyses for the association between every 1-h/day increment in ST and the composite endpoint of cardiovascular disease (stroke or myocardial infarction) or all-cause mortality. All HRs, CIs, and *P* values were derived from Cox regression models adjusted for sex and accelerometer wear time, as well as mutual adjustment for the other significant risk factors. *CI* confidence interval, *HR* hazard ratio, *IR* incidence rate, *PA* physical activity, *PY* person years, *ST* sedentary time

Every 1-h/day increment in ST was associated with 43% increased risk of CVD or mortality (HR 1.43, 95% CI 1.22–1.68) when adjusted for sex and accelerometer wear time, and 33% increased risk in the finally adjusted model (HR 1.33, 95% CI 1.14–1.56, Table 2). As shown in the subgroup analyses (Fig. 3), the association between ST and the risk of CVD or mortality was modified by MPA (*P* = 0.034 for interaction). Specifically, while every 1-h/day increment in ST was associated with 29% increased risk of CVD or mortality among participants performing ≤ 15 min/day of MPA (HR 1.29, 95% CI 1.01–1.65), the association was weakened for participants performing 16–29 min/day of MPA (HR 1.20, 95% CI 0.86–1.69) and further attenuated for participants performing at least 30 min/day of MPA (HR 1.11, 95% CI 0.82–1.50). Furthermore, the association between ST and the risk of CVD or mortality was stronger in current smokers (HR 2.05, 95% CI 1.24–3.38) than in non-smokers (HR 1.26, 95% CI 1.07–1.49) (*P* = 0.014 for interaction, Fig. 3).

### Sensitivity Analysis

After excluding all participants with a history of CVD and all participants with a follow-up time of 6 months or less, a total of 2779 participants and 97 events of CVD or mortality (stroke 25; MI, 26; death, 46) were included in the sensitivity analysis. The results of this analysis were similar to the results in the main analysis. Specifically, neither the association between every 30-min/day of LPA and the risk of CVD or mortality (HR 0.88, 95% CI 0.80–0.98), nor the association between every 1-h/day of ST and the risk of CVD or mortality (HR 1.32, 95% CI 1.10–1.59) were altered after adjusting for sex, accelerometer wear time, smoking, socioeconomic factors, medications and cardiometabolic risk factors. The association between every 30-min/day of MPA and the risk of CVD or mortality was only slightly weakened (HR 0.70, 95% CI 0.52–0.96).

## Discussion

In this prospective cohort study of 70-year-old individuals, we observed that greater amounts of objectively measured LPA and MPA were each associated with lower risk of stroke, MI or all-cause mortality, while greater amounts of ST were associated with increased risk of the outcomes. The associations were linear and independent of sex, accelerometer wear time, smoking, socioeconomic status, pre-existing CVD, medications, and cardiometabolic risk factors. Converted to 30 min/day increments, LPA was associated with 11% lower risk of CVD or mortality, and MPA was associated with 36% lower risk of CVD or mortality. In contrast, every 1-h/day increment in ST was associated with 33% increased risk of CVD or mortality.

### Outcomes

Earlier PA recommendations have primarily emphasized the importance of performing PA of at least moderate intensity; however, there is now an increasing interest in the potential benefits associated with LPA [16]. An emerging body of evidence indicate that LPA is associated with improved cardiometabolic markers, lower risk of CVD, and lower risk of premature death [23, 24, 33–36]. One of the key findings of the present study is that every 30-min/day increment in LPA was associated with 11% lower risk of CVD or mortality, even after adjusting for a vast number of potential confounders and mediators. This is in contrast to previous studies which have reported no benefits [25, 26], although supported by two previous studies showing similar estimates of risk reduction [23, 24]. Whereas these studies included only women with a higher mean age, our findings add an important piece to the puzzle by suggesting that LPA may have important benefits in both older men and women and of slightly younger age. Given that older people spend a majority of their awake time performing LPA and being sedentary [19, 37], these findings may have important implications from a public health perspective.

However, the results of the present study suggest that the largest estimated relative risk reduction is achieved through MPA. Converted to a 30-min/day increment, we found that MPA was associated with 36% lower risk of CVD or mortality, which extends the findings from previous studies [23, 26]. Of interest is that MPA showed a stronger association with CVD or mortality than LPA did, which with the limitation of being an observational study may indicate that higher intensity PA could be more beneficial with respect to prevention of CVD in the older individual. In light of this, it is of interest that in two randomized controlled trials conducted in similar populations, we found that 10 weeks of high-intensity exercise had positive effects on cardiovascular risk factors in 70-year-old men and women [38–40].

We also analyzed the associations of LPA and MPA in subgroups stratified based on amount of ST. Because the stratification resulted in smaller sample sizes, these are exploratory and preliminary findings which should be cautiously interpreted, and which require replication in larger studies before conclusions can be drawn. Nevertheless, they did reveal some interesting findings. Greater LPA and MPA were each associated with lower risk of CVD or mortality in older adults who were sedentary for around 9 h/day or more, but not in those with less ST. In this sense, it should be noted that the more sedentary half of our population is likely most representative of the general older individual [41, 42], and the findings could be interpreted as a reinforcement that as people age and become more sedentary, the importance of PA increases [41, 42].

The impact of sedentary behavior has gained increased attention during recent years and may be particularly important to study in older populations given the demographic shift towards more older people in our society [6]. In the present study, we observed a 33% increased risk of CVD or mortality for every 1-h/day increment in ST, which is considerably higher than the 12% risk estimate in a recent study in older women [43]. However, a perhaps even more important and interesting finding was that increased levels of MPA appeared to attenuate some of the risks associated with sedentary behavior. Already at a cut-point of 15 min/day of MPA, the association of ST with CVD or mortality was weaker, and the association was further weakened in participants with at least 30 min/day of MPA. These findings stand in contrast to a previous study on older men [44], which showed that adhering to the PA recommendations is not sufficient to attenuate the association of ST with mortality. However, the phenomenon has some support from previous meta-analyses which showed that 60–75 min of self-reported MPA/day diminishes the risk of mortality in sedentary individuals [45, 46]. However, as mentioned in the previous paragraph, the results of these subgroup analyses are preliminary and need to be replicated in larger studies. Because ST constitutes a large proportion of older peoples’ daily activity, it would have important implications for clinical practice if it can be established that MPA can attenuate some of the increased risks of CVD or mortality pertaining to prolonged ST [21, 22].

### Limitations and Strengths

There are some methodological aspects that need to be addressed. First, the present study had an observational design, meaning that causality cannot be established. There were also relatively few outcome events during follow-up, which may have influenced the statistical power of the analyses. In particular, this could be the case for the stratified analyses, and therefore, these are rather exploratory findings which require further confirmation. Still, there was sufficient power for the performed main analyses of PA and ST in relation to the outcomes. Furthermore, when studying PA in older adults in relation to incident disease, there is a risk of bias from reverse causation, so that the low PA at baseline in some participants may be due to early onset, or previous history of disease, as opposed to volitional inactivity, creating the association of PA with CVD. In an attempt to decrease this risk of bias, we excluded all participants with a history of CVD and with a short follow-up in a sensitivity analysis. As shown, the associations were only marginally altered. However, it is possible that some residual confounding still remains, as a recent paper suggested that the first 5 years of follow-up should be excluded to minimize reverse-causality bias [47]. Another methodological aspect to consider is the extent to which a single PA assessment represents “true” patterns of activity. A previous study showed that a 7-day assessment provided reliable estimates of PA and ST in older women over a period of 2–3 years [48]. It should, however, be noted that biased estimates of PA among the participants would likely attenuate all associations towards zero. Next, while there is no consensus on cut-points for intensity thresholds, the cut-points used in the present study are the most frequently utilized ones in studies on community-dwelling older adults, with the advantage of facilitating inter-study comparisons [49]. However, it should be noted that these were originally validated in younger adults [28], which would translate into higher relative intensities in the present cohort of 70-year-olds. Yet, the results showed that by using these cut-points, both LPA and MPA was associated with lower risk of CVD or mortality in our cohort of older men and women. Interestingly, similar associations were found in a recent meta-analysis on both middle-aged and older adults using same cut-points for intensity classification [33].

The present study also has several strengths. First, the use of accelerometers to objectively measure PA is an important strength which allows for accurate quantification of sedentary behavior and different intensities of PA as opposed to self-report measures [19, 20, 50]. For instance, in a previous study based on the same cohort, self-reported PA was more than twice that of accelerometer-derived measures of PA [51]. There was also a very high adherence to wearing the accelerometer, as more than 92% of the participants had sufficient wear time. Moreover, the study cohort which included over 3300 consecutive participants, with an equal distribution between women and men, was based on an ongoing population-based study with no exclusion criteria. There is, therefore, good potential for the findings to be generalized to other cohorts of community-dwelling older adults. We also adjusted for an extensive number of potential confounders and mediators thought to be in the causal pathway between PA and CVD, including smoking, socioeconomic factors, medical history, medications, and cardiometabolic risk factors. Altogether, these above-mentioned factors increase both the internal and external validity of the results.

## Conclusion

To summarize, this prospective study of more than 3300, 70-year-old men and women shows that objectively measured LPA and MPA are each associated with lower risk of stroke, MI or all-cause mortality, while ST is associated with increased risk of stroke, MI or all-cause mortality. In light of the rapid population ageing and persistent high burden of CVD in older people, our results may influence and support environmental actions and interventions aiming to reduce the risk of CVD and premature death in older adults by emphasizing the importance of promoting PA of all intensities and reducing ST. Finally, our findings can further be translated into three concrete messages that are easily communicated to the public: first, “regardless of intensity, the more you move and the less you sit, the better”. Second, “if you are able, aim to perform also MPA, such as brisk walking, as this may have even larger benefits”. Third, “by increasing the amount of MPA you may potentially mitigate some of the increased risks pertaining to sedentary behavior”.

## Declarations

### Data Availability Statement

The datasets analysed during the current study are not publicly available in accordance with the General Data Protection Regulation, but a de-identified data set may be available from the corresponding author on reasonable request.
